# Mycolactone Diffuses into the Peripheral Blood of Buruli Ulcer Patients - Implications for Diagnosis and Disease Monitoring

**DOI:** 10.1371/journal.pntd.0001237

**Published:** 2011-07-19

**Authors:** Fred S. Sarfo, Fabien Le Chevalier, N'Guetta Aka, Richard O. Phillips, Yaw Amoako, Ivo G. Boneca, Pascal Lenormand, Mireille Dosso, Mark Wansbrough-Jones, Romain Veyron-Churlet, Laure Guenin-Macé, Caroline Demangel

**Affiliations:** 1 Komfo Anokye Teaching Hospital, Kumasi, Ghana; 2 Institut Pasteur, Pathogénomique Mycobactérienne Intégrée, Paris, France; 3 Institut Pasteur, Mycobactéries Tuberculeuses et Atypiques, Abidjan, Côte d'Ivoire; 4 Kwame Nkrumah University of Science and Technology, Kumasi, Ghana; 5 Institut Pasteur, Biologie et Génétique de la Paroi Bactérienne, Paris, France; 6 INSERM, Groupe AVENIR, Paris, France; 7 Institut Pasteur, Plateforme Protéomique, Paris, France; 8 St George's University of London, London, United Kingdom; University of Tennessee, United States of America

## Abstract

**Background:**

*Mycobacterium ulcerans*, the causative agent of Buruli ulcer (BU), is unique among human pathogens in its capacity to produce a polyketide-derived macrolide called mycolactone, making this molecule an attractive candidate target for diagnosis and disease monitoring. Whether mycolactone diffuses from ulcerated lesions in clinically accessible samples and is modulated by antibiotic therapy remained to be established.

**Methodology/Principal Finding:**

Peripheral blood and ulcer exudates were sampled from patients at various stages of antibiotic therapy in Ghana and Ivory Coast. Total lipids were extracted from serum, white cell pellets and ulcer exudates with organic solvents. The presence of mycolactone in these extracts was then analyzed by a recently published, field-friendly method using thin layer chromatography and fluorescence detection. This approach did not allow us to detect mycolactone accurately, because of a high background due to co-extracted human lipids. We thus used a previously established approach based on high performance liquid chromatography coupled to mass spectrometry. By this means, we could identify structurally intact mycolactone in ulcer exudates and serum of patients, and evaluate the impact of antibiotic treatment on the concentration of mycolactone.

**Conclusions/Significance:**

Our study provides the proof of concept that assays based on mycolactone detection in serum and ulcer exudates can form the basis of BU diagnostic tests. However, the identification of mycolactone required a technology that is not compatible with field conditions and point-of-care assays for mycolactone detection remain to be worked out. Notably, we found mycolactone in ulcer exudates harvested at the end of antibiotic therapy, suggesting that the toxin is eliminated by BU patients at a slow rate. Our results also indicated that mycolactone titres in the serum may reflect a positive response to antibiotics, a possibility that it will be interesting to examine further through longitudinal studies.

## Author Summary

Mycolactone is a diffusible cytotoxin produced by *Mycobacterium ulcerans,* the causative agent of the skin disease Buruli ulcer. In a previous study using animal models, we reported that mycolactone released by cutaneous foci of infection gains access to the peripheral blood. Here we investigated whether mycolactone circulates in human patients and is detectable in clinically accessible samples. Using a combination of solvent extraction, high performance liquid chromatography and mass spectrometry analysis, we found that structurally intact mycolactone was present in ulcer exudates obtained from wound swabs and in the serum of patients. Unexpectedly, high titres of mycolactone were detected in ulcer exudates after completion of antibiotic treatment. In contrast, mycolactone could only be detected in the serum of newly diagnosed patients. Our results demonstrate that mycolactone detection in serum and ulcer exudates may be used to diagnose BU. Moreover, they suggest that the kinetics of mycolactone concentration in serum may be indicative of the clinical response of patients to antibiotic therapy.

## Introduction

Buruli ulcer (BU), caused by *Mycobacterium ulcerans*, is the third most common mycobacterial disease after tuberculosis and leprosy and represents an emerging threat [1], [2]. Since the late 1980s, the disease has been developing throughout West and Central Africa, prompting the WHO in 1998 to initiate an awareness and control campaign (http://www.who.int/gtb-buruli). Although efficient [3], [4], [5], [6], [7], current treatment protocols recommend the daily administration of oral rifampicin and intramuscular streptomycin for 8 weeks, with additional surgical intervention when necessary. To control the emergence of BU and to improve the management of the disease, it is vital to develop new tools for early diagnosis and treatment monitoring. A distinctive feature of *M. ulcerans* among human pathogens is the production of mycolactone [8], a macrocyclic polyketide playing a critical role in bacterial virulence (reviewed in [9], [10]). We have recently demonstrated the presence of intact mycolactone in punch biopsies from all forms of BU disease, before and during antibiotic therapy [11]. Moreover, there is evidence from mouse studies that mycolactone may diffuse into the peripheral blood [12]. Here we used chemical approaches to determine if mycolactone is present in blood samples and ulcer exudates obtained non-invasively at various stages of antibiotic therapy.

## Methods

### Patient cohorts

Patients were recruited if they met the WHO clinical case definition of BU disease; were not pregnant; had no history of tuberculosis, leprosy, or liver, kidney, or hearing impairment. All subjects provided written informed consent (thumb print of parent or guardian in the case of children, depending on literacy). A cross-section of patients with BU disease were recruited which included a spectrum of patients yet to initiate antibiotic therapy, some at various stages of antibiotic treatment and few who had completed treatment. Healthy controls from the same endemic area were included. In Ghana, patients were recruited by local health workers from villages near Tepa Government Hospital in the Ahafo Ano North District of Ghana, where there is a high prevalence of BU. The study protocol was approved by the ethics review committees at the School of Medical Sciences, Kwame Nkrumah University of Science and Technology, Kumasi, Ghana. In Ivory Coast, patients were either recruited from the Djekanou General Hospital or detected by a mobile medical team actively screening the district of Abidjan. The study protocol was approved by the national ethic review committee.

### Diagnosis and treatment

To confirm the clinical diagnosis, punch biopsy specimens of 4-mm diameter (Ghana) or ulcer exudates (Ivory Coast) were tested by PCR for the IS2404 repeat sequence, which is characteristic of *M. ulcerans*. Positive patients were treated with 10 mg/kg oral rifampicin and 15 mg/kg intramuscular streptomycin daily, administered at village health posts under direct observation, according to the WHO recommendations. Only IS2404 positive samples were considered for analysis of mycolactone presence.

### Mycolactone standard

Mycolactone was extracted from *M. ulcerans* 1615 (ATCC 35840), as previously described [12]. In brief, bacteria were cultivated in Middlebrook 7H9 broth (Difco) enriched with 10% oleic acid-albumin-dextrose-catalase (OADC, Becton Dickinson) for 4 weeks in spinner flasks at 30°C. Total lipids were extracted from bacterial cell pellets with 2/1 CHCl_3_/MeOH (v/v) for 20 h at 4°C. After separation from the aqueous phase following the addition of 20% H_2_O (w/v), the organic phase was dried. The resulting material was resuspended in ice-cold acetone and incubated for 20 h at −20°C. The acetone-soluble fraction was then dried, resuspended in ethanol, and loaded onto a silica gel TLC plate and eluted with 90/9/1 CHCl_3_/MeOH/H_2_O as the mobile phase. The yellow band corresponding to mycolactone (retention factor of 0.2) was then scraped and mycolactone eluted from silica particles using 2/1 CHCl_3_/MeOH (v/v). Following solvent evaporation, purified mycolactone was resuspended in ethanol. The concentration of the resulting solution was determined by UV absorption, as described [13].

### Mycolactone detection by coupling to 2-naphtalene boronic acid (TLC-Fluo)

As recently described [13], mycolactone or lipid extracts were applied to a silica gel TLC plate and eluted with 90/9/1 CHCl_3_/MeOH/H_2_O as the mobile phase. The eluted TLC plate was briefly warmed on a hot plate to evaporate the organic solvents, and quickly immersed into a 0.1 M acetone solution of 2-naphtalene boronic acid (Sigma), then heated to 100°C for 5∼10 seconds. The TLC plate was then irradiated with a UV reader equipped with a 312 nm lamp.

### Mycolactone detection by HPLC/MS/MS

Mycolactone or lipid extracts were also analyzed by High Performance Liquid Chromatography coupled to Mass Spectrometry (HPLC/MS/MS). We used a Shimadzu HPLC fitted with a BDS Hypersil C8 column (5 µm, 4.6×250 mm), with UV detection at 360 nm. Mycolactone was eluted by a 60 min gradient from 50 to 95% acetonitrile in water after 22 min. Fractions of interest were collected in glass tubes and analysed on a QSTAR XL, AB-MDS-SCIEX mass spectrometer with an electrospray ion source and the following parameters: ion spray voltage (IS), 5200 v; curtain gas (CUR), 25; gas 1 (GS1), 5; declustering potential (DP), 50 v; focusing potential (FP), 225 v; declustering potential 2 (DP2), 15 v. Mycolactone was identified by the presence of [M+Na]+ *m*/*z* 765.5. MS-MS parameters were: ion spray voltage (IS), 6000 v; curtain gas (CUR), 25; gas 1 (GS1), 20; declustering potential (DP), 60 v; focusing potential (FP), 230 v; declustering potential 2 (DP2), 10 v; collision energy (CE), 60; collision gaz (CAD), 5. Data were collected and processed through the Analyst QS 1.1 software from AB-MDS-Sciex.

### Exudates

Wound swabs obtained from the undermined edges of ulcerative lesions were soaked into 1 ml ethanol immediately after sampling and stored in polypropylene collecting tubes at -20°C, protected from light, until analysis. Ethanol solutions of exudates were concentrated to a volume of 500 µl then processed to TLC-Fluo and HPLC/MS/MS analyses.

### Serum samples

Lipids were extracted from serum samples (1 ml) by sequential addition of 4/1 MeOH (v/v), 1/1 CHCl_3_ (v/v), and 3/1 H_2_O (v/v), each step being followed by thorough mixing. The upper aqueous phase was discarded and the bottom organic phase transferred to a glass tube containing 3 ml of MeOH. The resulting solution was centrifuged to sediment insoluble particulate matter. The soluble organic phase was then dried and the resulting product re-suspended in ethanol for TLC-Fluo and HPLC/MS/MS analyses.

### Mononuclear cell pellets

Mononuclear cells were isolated from 10 ml whole blood by differential sedimentation on Ficoll-Hypaque (GE Healthcare). Cell pellets were then dried and stored at −20°C until lipid analysis. Total lipids were extracted from cell pellets by addition of 1 ml 2/1 CHCl_3_/MeOH (v/v) for 48 h at 4°C. The organic phase was recovered by addition of 20% H_2_O (w/v), dried and the resulting product resuspended in ethanol for TLC-Fluo and HPLC/MS/MS analyses.

## Results

### Efficacy of mycolactone extraction from biological samples

**Figure 1:**
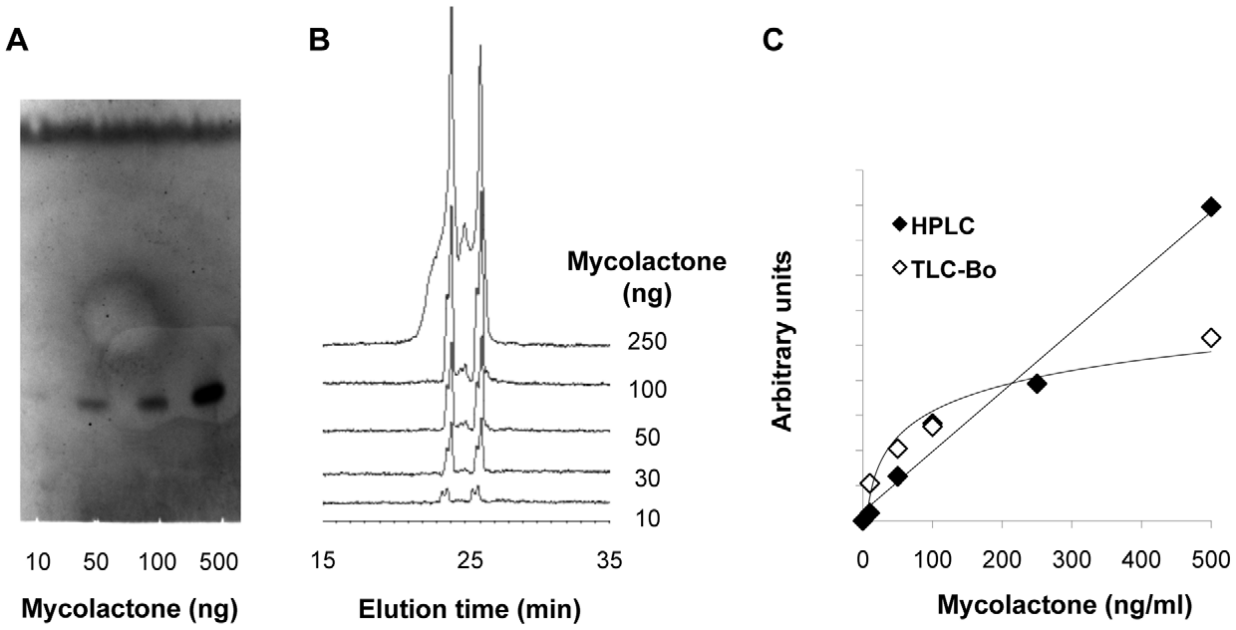
Comparison of the performances of the TLC-Fluo and HPLC approaches for mycolactone quantitative detection. A representative picture of the fluorescent signals and elution peaks obtained for mycolactone (10–500 ng) detection by TLC-Fluo (A) and HPLC (B) are shown, with the corresponding standard curves (C). Arbitrary units correspond to fluorescence intensity of the mycolactone band (TLC-Fluo) and area of the mycolactone elution peak (HPLC). Similar results were obtained in at least three independent experiments.

We first compared the TLC-Fluo and HPLC/MS/MS approaches for mycolactone detection in the 0–500 ng range, using purified mycolactone as a reference. Both methods were highly sensitive, yielding a detectable signal with only 10 ng mycolactone (Fig. 1A,B). However the fluorescent signal of mycolactone modified by coupling to 2-naphtalene boronic acid was not a linear function of its concentration, whereas the areas of mycolactone elution peaks in HPLC were proportional to mycolactone concentration in the 10–500 ng domain (Fig. 1C). To evaluate the efficacy of mycolactone extraction from biological samples, serum samples (1 ml) or mononuclear cell pellets (10^6^ cells) were spiked with purified mycolactone. We then proceeded to solvent extraction as described in the Methods section, and estimated the proportion of recovered mycolactone using the above-described standard curves. The estimated yields of extraction with organic solvents were of 20% from cell pellets and 10% from serum samples (not shown). Several combinations of solvents were tried that did not improve the recovery of mycolactone.

### Detection of mycolactone in ulcer exudates

**Figure 2:**
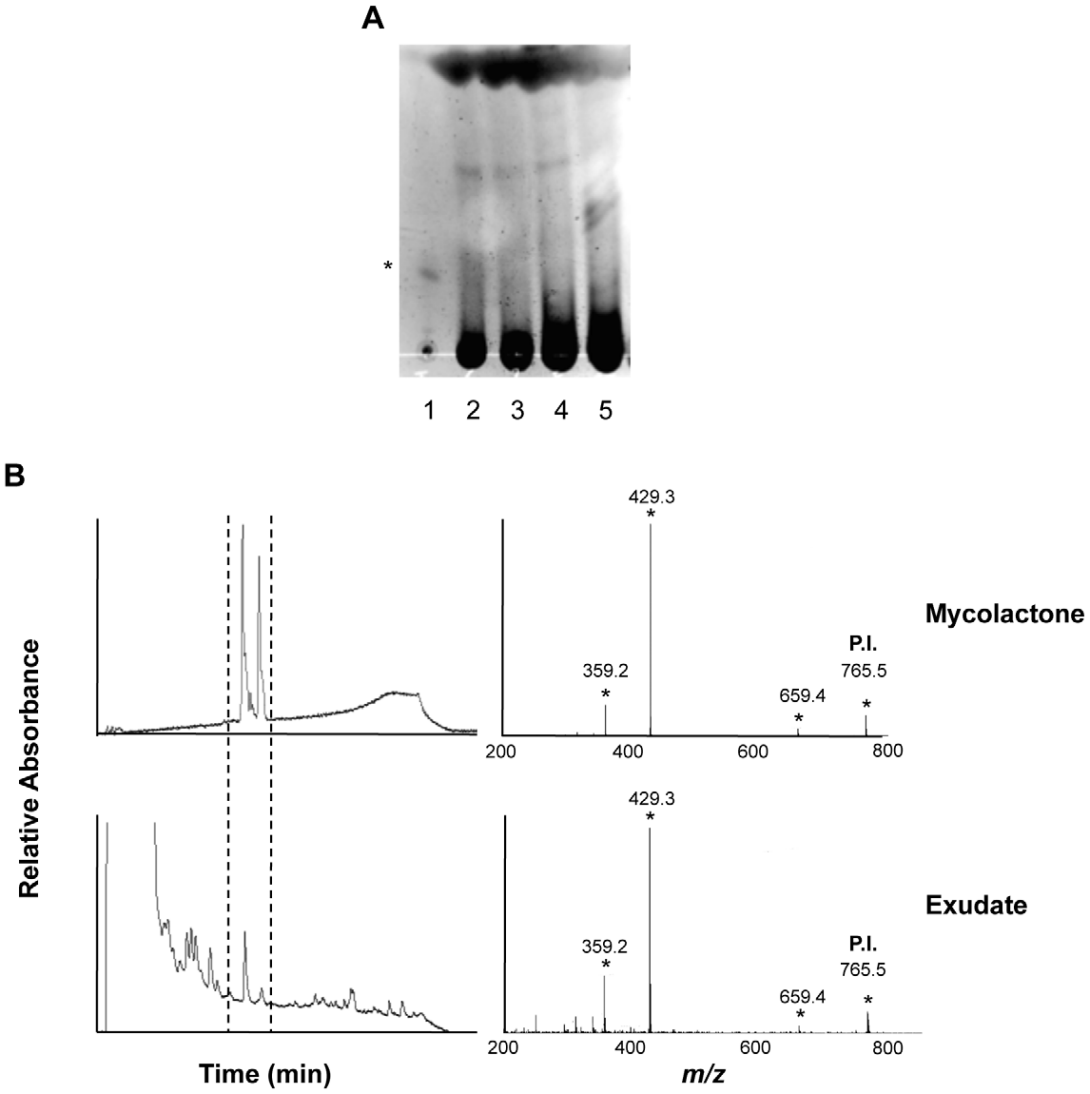
Structurally intact mycolactone is detected in ulcer exudates of BU patients. (A) Example of the fluorescence signals given by ulcer exudates (lanes 2–5), compared to pure mycolactone (lane 1) following analysis by TLC-Fluo. The band corresponding to mycolactone is designated by an asterisk. (B) HPLC elution profiles are shown for reference mycolactone (100 ng) and for one representative ulcer exudate among the 20 positive ones. The corresponding MS/MS spectra are presented, with mycolactone parent ion (P.I.) and products designated by asterisks. Similar MS/MS spectra were obtained from HPLC elution peaks collected from three positive patients.

**Figure 3:**
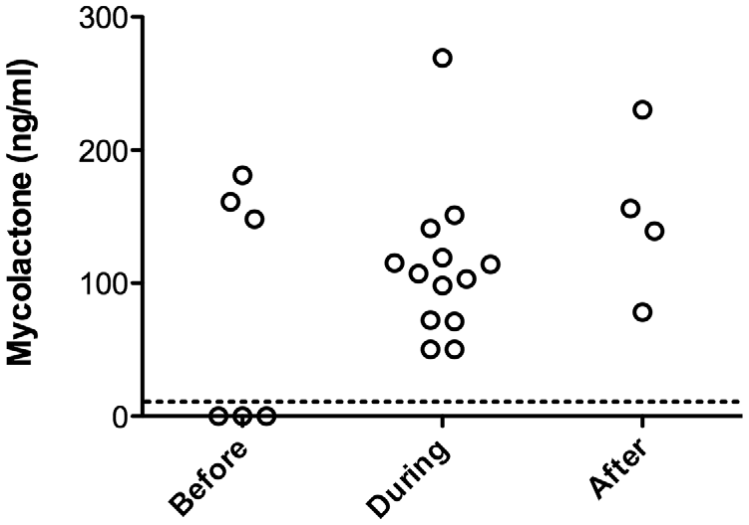
Mycolactone presence is maintained in ulcer exudates during antibiotic therapy. Mean concentration of mycolactone in ulcer exudates harvested before (0 week), during (2 to 8 weeks of treatment), or after completion of the 8 week antibiotic treatment. Dashed horizontal line indicates detection threshold.

Exudate samples were split into two equivalent parts, which were analyzed in parallel for the presence of mycolactone by HPLC/MS/MS or TLC-Fluo. We could not conclude on the presence of mycolactone by TLC-Fluo, because of the co-migration of auto-fluorescent compounds (Fig. 2A). Using HPLC determination, elution peaks corresponding to mycolactone were observed in 3/6 newly diagnosed patients and all patients undergoing or completing their course of antibiotic treatment (13/13, 4/4 respectively) (Table 1). To confirm that they effectively contained mycolactone, elution peaks were collected in three patients, and analyzed by MS/MS. In all of them, the characteristic spectrum of mycolactone parent ion (*m/z* 765) and products was observed [14], demonstrating the presence and structural integrity of mycolactone in ulcer exudates (Fig. 2B). Notably, the presence of mycolactone in these samples persisted during and after completion of antibiotic therapy (Fig. 3).

**Table 1:** Characteristics of patients and controls.

Ulcer exudates	Number	Age^a^	Gender (M/F)^b^	HPLC^c^
Befored	6	23.3	0/6	50
Duringe	13	16.7	7/6	100
Afterf	4	20.7	0/4	100
Ctrlg	NA	NA	NA	NA

Serum samples(Ghana)	Number	Age^a^	Gender (M/F)^b^	TLC-Bo^c^
Before^d^	8	13.1	5/3	50
During^e^	6	19.8	3/3	17
After^f^	4	29.2	2/2	25
Ctrl^g^	1	32.0	1/0	0

Serum samples(Ivory Coast)	Number	Age^a^	Gender (M/F)^b^	HPLC^c^
Befored	5	ND	ND	60
Duringe	8	23.5	1/3	12
Afterf	4	19.7	2/5	0
Ctrlg	3	ND	ND	0

Mononuclear cells(Ghana)	Number	Age^a^	Gender (M/F)^b^	HPLC^c^
Before^d^	15	21.1	9/6	0
Duringe	20	24.9	9/11	0
Afterf	7	24.7	4/3	0
Ctrlg	4	14.2	1/3	0

Mononuclear cells(Ivory Coast)	Number	Age^a^	Gender (M/F)^b^	HPLC^c^
Befored	3	33.3	1/2	0
Duringe	4	26.0	2/2	0
Afterf	5	19.6	0/5	0
Ctrlg	4	30.0	2/2	0

a Mean age (year).

b M, males; F, females.

c Percentage of samples with signal superior to background.

d Newly diagnosed.

e Treated for 2 to 8 weeks.

f Completed the treatment.

g Healthy control from the same endemic zone.

NA: not applicable; ND: not determined.

### Detection of mycolactone in blood mononuclear cells

Since our previous experiments in experimentally infected mice demonstrated the presence of mycolactone in circulating mononuclear cells, we analyzed this biological material in BU patients. Whole blood (10 ml) was collected in patients from Ghana and Ivory Coast at various stages of the disease (Table 1) and mononuclear cells were isolated by Ficoll gradient centrifugation. However, no mycolactone could be identified in any of the samples tested (n = 52) by TLC-Fluo or HPLC/MS/MS (not shown).

### Detection of mycolactone in serum

**Figure 4:**
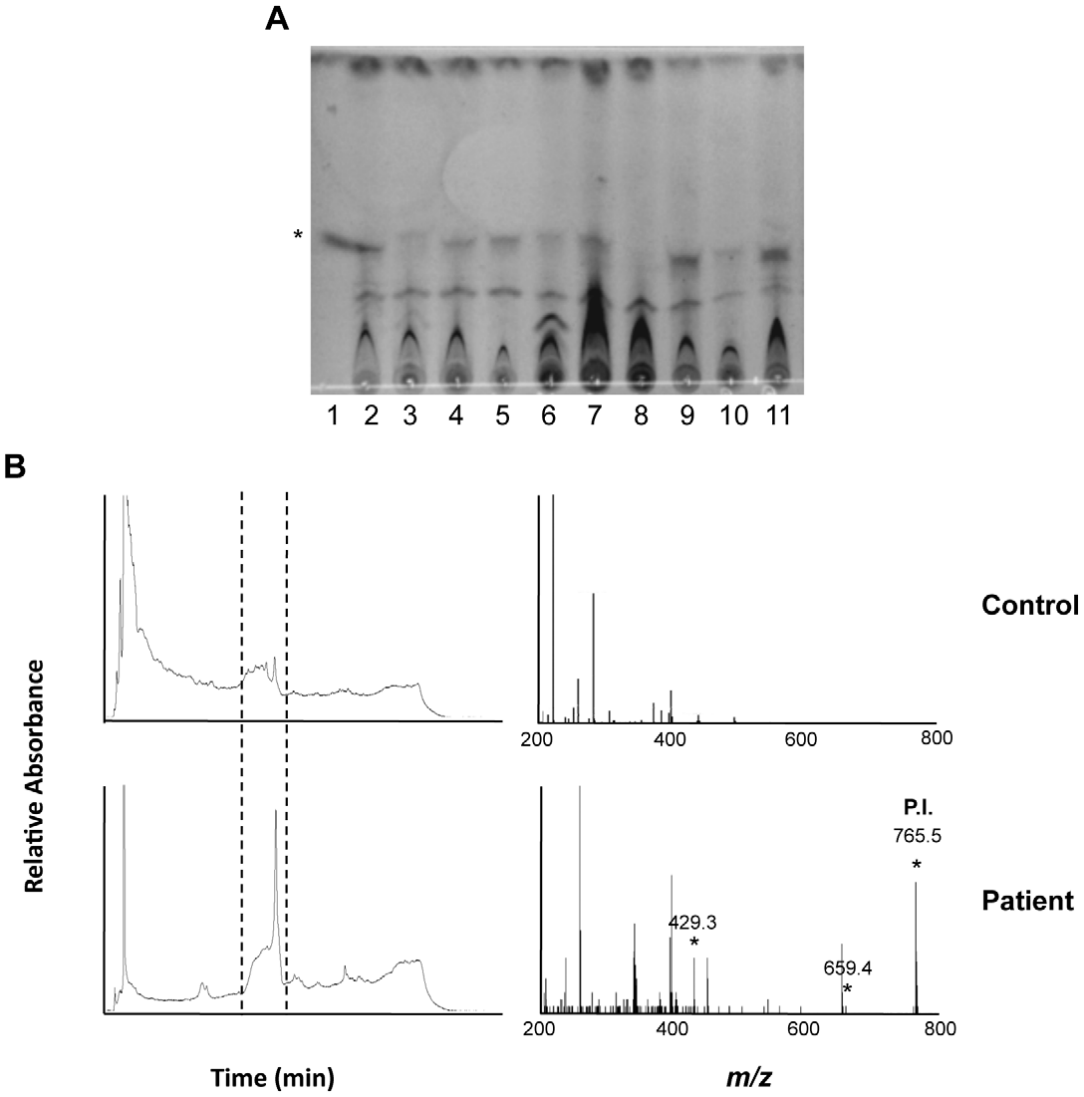
Structurally intact mycolactone is detected in serum samples of BU patients. (A) Example of the fluorescence signals given by serum samples (lanes 2–9), following lipid extraction and analysis by TLC-Fluo. Controls include 1 µg pure mycolactone (lane 1), lipids extracted from a negative serum (lane 10), and then spiked with 1 µg mycolactone (lane 11). The band corresponding to mycolactone is designated by an asterisk. (B) Representative HPLC elution profiles of lipids extracted from serum samples are shown for one healthy control out of 5, and one BU patient among the 4 positive ones. The corresponding MS/MS spectra show the presence of the parent and product ions of mycolactone in this positive sample. Similar results were obtained in a second one.

**Figure 5:**
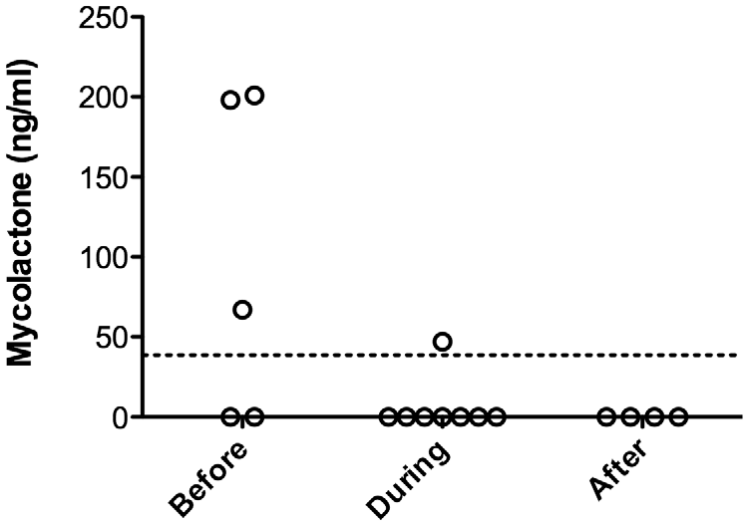
Mycolactone concentration in the serum of BU patients during antibiotic therapy. Mean concentration of mycolactone in serum samples collected before (0 week), during (2 to 8 weeks of treatment), or after completion of the 8 week antibiotic treatment. Dashed horizontal line indicates detection threshold.

Total lipids were extracted from 1 ml serum samples then analyzed by TLC-Fluo (Ghana samples) or HPLC (Ivory Coast samples) (Table 1). Again, the identification of mycolactone by TLC-Fluo was difficult, because of the co-migration of auto-fluorescent compounds (Fig. 4A). Using HPLC, mycolactone detection was also hampered by the co-elution of UV-absorbing contaminants (Fig. 4B). We could nevertheless identify mycolactone-like peaks in 3/5 newly diagnosed patients, 1/8 patients undergoing treatment, and 0/4 patients completing treatment. These peaks were collected in two patients and analyzed by MS/MS. In both of them, the characteristic spectrum of mycolactone parent and product ions could be observed (Fig. 4B). The concentration of circulating mycolactone was evaluated in positive samples by measuring the area of mycolactone elution peaks and subtracting the mean signal of two healthy controls (Fig. 5). Calculated values were in the 40–200 ng/ml range.

## Discussion

In the present study, we investigated whether mycolactone was detectable in easily accessible samples of BU patients. We used two chemical approaches, both requiring the extraction of total lipids by organic solvents. The efficacy of this extraction step, as measured by addition of pure mycolactone to control samples, was mediocre and reduced dramatically the sensitivity of the following TLC-Fluo and HPLC determinations. An explanation for such a limited yield of extraction may be that mycolactone associates with biomolecules preventing solvent access, a possibility that we are currently testing by studying the impact of various thermal and enzymatic treatments. If the efficacy and selectivity of mycolactone extraction can be improved, the recently described and field-friendly TLC-Fluo detection method may still be an option for mycolactone-based point-of-care diagnostic tests. If not, it will be necessary to design alternative approaches that do not require this purification step.

Using HPLC/MS/MS, we could demonstrate that mycolactone gains access to the peripheral blood of human patients. In previous studies in the mouse model, we detected structurally intact mycolactone in mononuclear cell fractions of pooled blood samples harvested from mice subcutaneously injected with mycolactone, or experimentally infected with *M. ulcerans* [12]. In the present work, mycolactone could not be identified in blood mononuclear cells. This cell subpopulation was isolated from 10 ml whole blood, and we estimated the maximum yield of mycolactone extraction from mononuclear cell pellets to 20%. If mycolactone effectively reaches blood mononuclear cells in human patients, its cellular concentration may be too low to be detected in the accessible volume of blood. Alternatively, mycolactone may be unstable in the conditions used in the present study to isolate and store mononuclear cell pellets.

In contrast, we were able to demonstrate the presence of structurally intact mycolactone in the serum of 3/5 newly diagnosed BU patients (Table 1). This novel information provides the essential proof of concept for the design of BU diagnostic tests based on mycolactone detection in peripheral blood. Since mycolactone is extracted from serum samples with low efficacy, the number of positive samples and the calculated concentrations of circulating mycolactone are probably underestimated in this study. Whether mycolactone kinetics in serum could be employed to monitor the response to antibiotic treatment will certainly be interesting to investigate further. Our preliminary results suggest that, in spite of a sustained presence in ulcer exudates (Fig. 3), mycolactone concentration (Fig. 5) showed a tendency to decrease in the serum during antibiotic therapy. If confirmed by longitudinal studies, the decay of circulating mycolactone during antibiotic therapy would provide an explanation to the recovery of cellular immune responses during treatment [15], [16] and after surgical excision of BU lesions [17].

Here we considered patients at ulcerative stages of the disease. BU is usually diagnosed on the basis of clinical symptoms, as the identification of *M. ulcerans* by means of cultures or PCR requires dedicated facilities and specialized equipment (reviewed in [18]). Common differential diagnoses of BU include other tropical ulcers (venous, phagedenic, neurogenic), leishmaniasis, yaws and squamous cell carcinoma. The presence of biologically active mycolactone was recently demonstrated in skin biopsies of BU patients [11]. Here we show that mycolactone can be detected in ulcer exudates obtained non-invasively from wound swabs, in a structurally intact form and at concentrations in the 50–200 ng/ml range, which strongly suggests that mycolactone detection in exudates may be of interest for differential diagnosis of the ulcerative forms. Whether mycolactone is present at pre-ulcerative stages in fine-needle aspirates and in the peripheral blood is currently under investigation.

We observed significant amounts of mycolactone in exudates at the end of antibiotic therapy. In some instances, antibiotic pressure may not efficiently block, and could even enhance the production of a toxin. For instance, in patients infected with *E*. *coli* O157*∶*H7, the use of antimicrobials has been discouraged because it stimulates toxin production and augments the risk of detrimental, or even fatal complications [19]. Whether antibiotics promote the expression of mycolactone by *M. ulcerans* is unknown. However, their efficacy at killing *M. ulcerans* is not questionable with 4 weeks of treatment leading to culture negativity [3], [4], [5], [6], [7]. Although a stimulatory effect of antibiotics on mycolactone production cannot be excluded, our observation suggests that mycolactone persists in cutaneous tissues after the demise of *M. ulcerans*. Since mycolactone displays inherent ulcerative properties [8], this phenomenon may explain why some BU take considerable time to heal despite remaining culture negative.

The administration of antibiotics is sometimes associated with adverse reactions, excessive inflammation and pain sensation, that paradoxically make the symptoms of infection worse [20]. It is possible that in BU, like in the Jarish-Heixheimer reaction in syphilis or in relapsing fevers, efficient killing of *M. ulcerans* by antibiotics leads to the sudden and massive release of bacterial antigens locally that act as immuno-stimulants. Mycolactone displays potent immunosuppressive properties *in vitro* that are thought to contribute to the cellular response defects of BU patients [21], [22], [23], [24], [25]. The rapid decline of mycolactone from the systemic circulation during treatment may thus provoke exuberant inflammatory responses at the level of the lesions, and cause the paradoxical reactions observed in BU disease [26].
